# An Underlooked Cause of Periodic Fever (PFAPA) in an Adult Patient with No Response to Tonsillectomy

**DOI:** 10.1155/2018/6580835

**Published:** 2018-05-02

**Authors:** Fiaz Alam, Mohammed Hammoudeh

**Affiliations:** Department of Medicine, Hamad General Hospital, Doha, Qatar

## Abstract

Periodic fever with aphthous stomatitis, pharyngitis, and adenitis (PFAPA) is an autoinflammatory disease manifested as recurrent febrile episodes associated with one of the following cardinal features: aphthous ulceration, pharyngitis, and cervical adenitis. It was initially described in children and thought to be a disease of pediatric age group. Few adult cases were also reported in the literature. We describe the case of a 39-year-old female affected by PFAPA who presented with a history of febrile episodes associated with aphthous ulceration, stomatitis, and tonsillitis for 4 years. The febrile episodes occurred at a regular interval of 4 weeks and resolved within 5 days. The patient underwent tonsillectomy without any significant improvement. The patient responded only to a single high dose of steroid during the attack. Although PFAPA was initially thought to be a disease of pediatric age group, it should be considered in patients with recurrent febrile illness in all age groups.

## 1. Introduction

Periodic fever syndrome or autoinflammatory diseases are group of disorders characterized by recurrent episodes of systemic inflammation. PFAPA is among one of these chronic autoinflammatory diseases, which is diagnosed on clinical ground. It is manifested as recurrent episodes of fever associated with tonsillitis, aphthous ulceration, stomatitis, and cervical adenitis. It is initially described in children by Marshall in 1987 [1, 2].

Classical presentation of PFAPA is recurrent episodes of fever, which occurs at intervals of 3 to 5 weeks associated with one of the following cardinal signs: aphthous ulceration, stomatitis, tonsillitis, and cervical adenitis [3, 4].

Children aged between 2 and 5 years are most commonly affected. The exact cause is not known, but rapid response to the single high dose of steroid suggests autoinflammatory mechanism [5].

## 2. Case Presentation

A 39-year-old lady was referred to a rheumatology clinic with a history of recurrent febrile illness. The patient was enjoying her usual life till the age of 35 years when she developed fever associated with aphthous ulcers, stomatitis, and tonsillitis. The patient was also having severe myalgia, arthralgia, and generalized body aches to an extent that she was unable to carry out her daily activities. This episode resolved after five days. Thereafter, the patient had recurrent similar febrile episodes every 4th week. The patient was seen by her physician, and initially, she was managed as having viral infection and then as tonsillitis with antibiotics. She continued to have similar episodes at regular intervals. The attacks were so regular that the patient and her family could foretell the exact day when an episode will occur. The patient underwent tonsillectomy as she was having regular febrile episodes with tonsillitis and stomatitis for almost 2 years. After tonsillectomy, she continued to have similar febrile episodes at regular intervals but with less intensity and less frequency for almost 6 months. Six months after surgery, the patient started to have similar attacks with increase in severity. At her last febrile episode, the patient was admitted to a hospital and extensively investigated as a case of pyrexia of unknown origin. The following set of investigations were all normal during her stay in hospital: full blood count, renal function, liver function, urine and blood cultures, swab culture from throat, ASO titers, and CT imaging of neck, chest, abdomen, and pelvis. The patient was discharged with outpatient referral to the rheumatology department.

In the rheumatology outpatient clinic, the patient was initially seen between the attacks when she was completely asymptomatic. A detailed history was taken from the patient. There was no history of joint swelling, skin rash, hair fall, Raynaud's phenomenon, and genital ulceration. Systemic review was unremarkable. There was no history of headache, recurrent chest pain, shortness of breath, recurrent abdominal pain, or urinary symptoms. Between the episodes, the patient was completely normal and living a healthy life. Febrile episode was not related to the menstrual cycle of patient.

There was no history of similar recurrent febrile episode in any of the first- or second-degree family member.

On examination, the patient was not in any apparent distress and looked well built.

Her weight was 68 kg, temperature 37°C, blood pressure 114/70, and pulse 82/min (regular).

The patient was not pale and not jaundiced. There was no cervical and axillary lymphadenopathy. Ear and nose examination were normal. Oral cavity examination was completely normal. Lung and heart examination revealed normal vesicular breathing and normal heart sounds, respectively. Abdominal examination was also normal with no evidence of organomegaly. Further investigation revealed negative ANA, rheumatoid factor, and negative antibodies against extractable nuclear antigen (ENA).

Clinical diagnosis of PFAPA was made, and the patient was started on colchicine 0.5 mg orally twice daily and later on ranitidine 150 mg once daily as prophylaxis, but she continued to have similar attacks every 4 to 5 weeks.

The patient was advised to take prednisolone 60 mg orally with one dose at the start of episode.

The patient responded to the single high dose of steroid with resolution of symptoms within 1 day. On regular follow-up, the patient did report of more frequent attacks every 2 to 3 weeks after starting steroid. Currently, the patient is advised to take a single high dose of prednisolone at the start of febrile illness. The patient lost follow-up as she moved to a different country.

## 3. Discussion

We are presenting an adult female who developed PFAPA disease at age 35 and was diagnosed at age 39.

PFAPA is one of the common causes of recurrent febrile illness in children. Common age group affected by this illness is between 2 and 5 years [3, 4]. It usually settles down before puberty. Only minority of children continued to have febrile episodes in their adulthood.

Kyvsgaard et al. [3] reported that the mean age of this febrile illness is 33 months. The mean duration of fever episodes was 4.45 days, and the mean duration of intervals between fever episodes was 29.66 days. In their study, erythematous pharyngitis was present in 80.6% of the patients, exudative pharyngitis in 61.3% of the patients, aphthous stomatitis in 48.4% of the patients, and cervical adenitis in 96.8% of the patients.

Feder and Salazar [4] included one hundred five children who met the criteria for PFAPA syndrome in their study. Accompanying signs and symptoms included aphthous stomatitis (38%), pharyngitis (85%), and cervical adenitis (62%).

In contrast to this, growing evidence suggests that PFAPA is not only limited to pediatric age group but can manifest in adulthood as well.

Mean age of 20 ± 7.5 was reported by Padeh et al. [6] in his retrospective study of 15 patients diagnosed with PFAPA with delay in diagnosis of more than 6 years. All patients in that study were having recurrent febrile illness associated with tonsillitis and cervical adenitis. One patient had tonsillectomy after the febrile episode but did not improve.

Cantarini et al. [7] reported 17 cases of adults who fulfilled the clinical criteria of PFAPA syndrome. The mean age of onset for recurrent episodes was 25.9 ± 8.3 years, and the mean duration was 5.5 ± 1.8 days. Similar to our patient, 10 out of 17 had combinations of 2 signs out of 3 cardinal signs (aphthous stomatitis, pharyngitis, and lymphadenitis).

Our patient has onset of febrile episode at 35 years, which is quite unusual for PFAPA syndrome. There was a delay of four years before diagnosing her as a case of PFAPA. The patient was completely normal between the attacks. Attacks were so regular that the patient and her family were quite sure about the day at which the episode will kick off. Attacks were typically associated with tonsillitis and aphthous ulceration and stomatitis. Our patient did not have adenitis.

Some of the earlier studies reported cases of PFAPA in adults younger than 30 years of age. Late presentation and diagnosis of PFAPA, like in our patient, was reported by Vitale et al. [8], where the mean age at disease onset was 33.75 ± 14.01 years and the mean age at diagnosis was 39.1 ± 14.39 years.

Tonsillectomy is considered as one of the treatment options for patients suffering from PFAPA syndrome.

Various studies [9, 10] have shown promising outcome after surgery with complete resolution of symptoms in children.

PFAPA syndrome is not frequently reported in adults, and the effect of tonsillectomy is not studied well. Cantarini et al. [7] reported 17 adult patients with PFAPA syndrome. Only two out of nine patients, who underwent tonsillectomy, showed clinical response.

Data from the literature [5] show that tonsillectomy was performed in 16 out of 36 adult patients diagnosed with PFAPA syndrome: complete response to surgery was seen in 1 patient, partial response in 2 patients, and no response in 13 patients. Out of 16 patients, 4 had tonsillectomy performed during infancy due to recurrent febrile pharyngitis. Three out of those 4 patients achieved disease-free period for several years before the recurrence of fever episodes.

Our patient underwent tonsillectomy with decrease in severity and frequency of episodes that lasted only for 6 months. Thereafter, regular febrile episodes continued in the form of fever, aphthous stomatitis, and pharyngitis associated with arthralgia and myalgia. This indicates that tonsillectomy did not cause resolution of symptoms of PFAPA. Its role is controversial especially in adult patients. There is no convincing evidence in the literature to explain why tonsillectomy is more effective in children than adults. Colotto et al. [12] reported that the effect of tonsillectomy could be transient, and febrile episodes could occur several years after the resolution of symptoms after tonsillectomy. The initial response could be due to removal of active lymphoid tissue, which could be the cause of immune dysregulation in PFAPA. However, the febrile episode could recur due to compensatory hypertrophy of other oral lymphoid reservoirs.

Corticosteroids [5, 10, 11] are effective in almost every child with PFAPA syndrome but comparatively less effective in adult patients.

Our patient did respond to high-dose prednisolone. The beneficial effect of single dose of steroid has been well established in various studies. At the same time, our patient did experience more frequent attacks with shortening of attack-free interval.

Cantarini et al. [7] reported that 11 out of 14 adult patients with PFAPA responded to corticosteroid therapy. Prednisone at the dose of 50–60  mg/day was administered in 33/36 patients, with a complete response in 28, partial response in 4, and ineffectiveness in 1.

Shortening of symptom-free interval [5, 10] (both in pediatric and adult patients) has been reported in the literature. Nine out of the 33 adults [5] treated with steroid experienced shortening of a symptom-free interval.

To avoid periodic attacks of fever, different medications have been tried as prophylactic medication. Colchicine and cimetidine [5, 13] are the two drugs used to prevent recurrent attacks of febrile illness with some beneficial effects, which was not the case in our patient.

PFAPA is initially thought to be the disease of pediatric age group, but cases in adults had also been reported. It is of utmost importance to be aware of this relatively benign cause of recurrent febrile episodes, as it will help in early diagnosis and prompt treatment and will also be a sign of relief for the patient and the physician as well. Effect of tonsillectomy might be transient and probably is not the treatment of choice for patients with PFAPA syndrome in adults, and more data are required to explain the role of tonsillectomy in adult patients with PFAPA syndrome.
